# A Rare Association of Congenital Diaphragmatic Hernia with Lower Esophageal Atresia and Perforation

**DOI:** 10.1155/2010/738546

**Published:** 2010-07-18

**Authors:** Narendra Kumar Are, K. Nagarjuna, Lavanya Kannaiyan

**Affiliations:** Department of Paediatric Surgery, Niloufer Hospital for Women and Children, Osmania Medical College, Hyderabad 500 017, India

## Abstract

Congenital diaphragmatic hernia is known to be associated with esophageal atresia, which is a rare association. We report a rare occurrence of congenital diaphragmatic hernia and lower esophageal atresia.

## 1. Introduction

Abnormalities of the esophagus such as gastroesophageal reflux disease (GERD), esophageal motility disorders, esophageal duplications cysts, and tracheoesophageal fistula with esophageal atresia are rare but documented occurrences with congenital diaphragmatic hernia [1, 2]. We report a rare association of lower esophageal atresia with congenital diaphragmatic hernia (CDH).

## 2. Case Report

**Figure 1:**
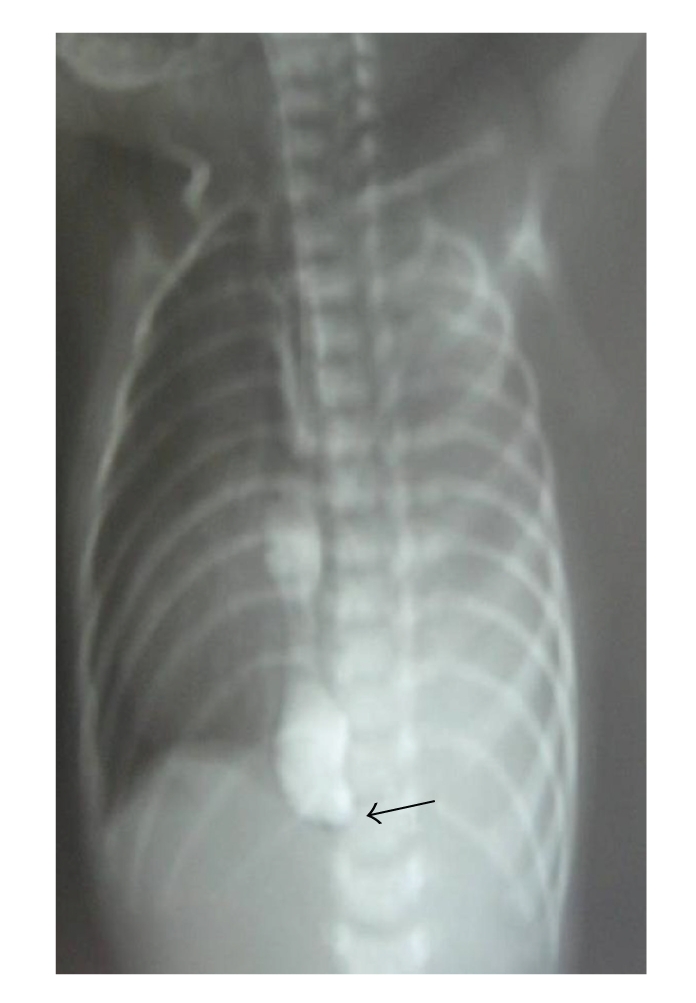
Contrast esophagogram showing holding up (arrow) of contrast at the level of the diaphragm with evidence of CDH with lower esophageal obstruction.

**Figure 2:**
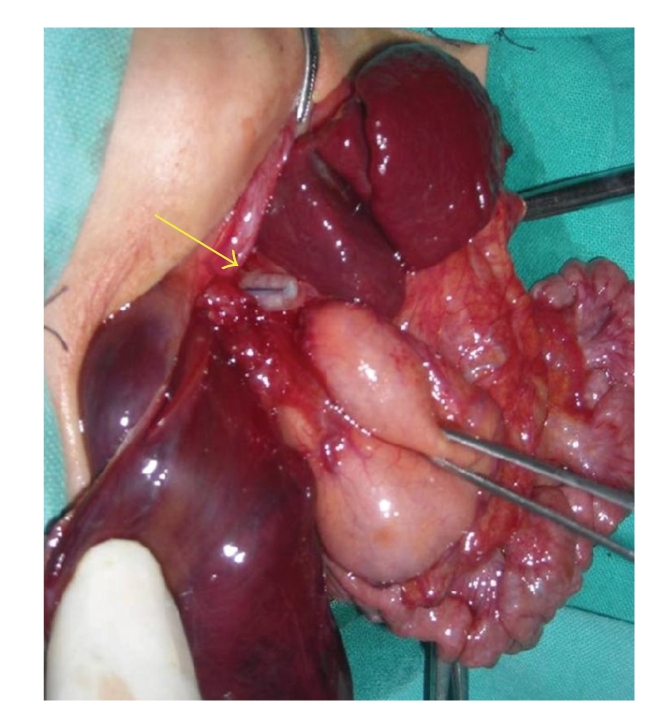
Operative photograph showing a blind ending of the lower esophagus with a sealed perforation (arrow).

A 10-day male child presented with respiratory distress without cyanosis since birth. On clinical examination, patient had tachypnea, with a scaphoid abdomen. The persistent drooling of saliva led to the suspicion of esophageal atresia. A red rubber catheter was passed into the esophagus, but there was resistance at 15 cm from the alveolar margin. Chest X-ray showed evidence of left CDH with mediastinal shift and the tip of nasogastric tube at the level of the diaphragm. A contrast esophagogram was done which showed holdup of dye at the level of the diaphragm (Figure 1). With the suspicion of associated esophageal obstruction and CDH, a laparotomy was done using a chevron incision. The operative findings include left posterolateral CDH, complete disruption of the esophagogastric junction with a blind-ending esophagus, and a sealed esophageal perforation at the esophagogastric junction (Figure 2).

The surgical correction included repair of CDH and esophagogastric anastomosis with a feeding jejunostomy. The postoperative course was uneventful. Jejunostomy feeds were started on the 4th postoperative day. Contrast esophagogram was done on the 10th postoperative day. It showed free flow of dye into the stomach. At discharge, the child was on full oral feeds. He has been followed up for 3 months. The child's general condition is good with adequate weight gain.

## 3. Discussion

Esophageal anomalies are known to be associated with CDH. These associations include tracheoesophageal fistula with esophageal atresia, GERD, esophageal dysmotility, esophageal duplication cysts, and esophageal ectasia [1, 2]. The possible noted explanations include the kinking of the esophagogastric junction [2] and abnormal innervation of the esophagus by the vagus and recurrent laryngeal nerve [3].

Tracheoesophageal fistula with esophageal atresia is a known association of CDH, albeit a rare one with an incidence of 0.005 per 1000 births [4]. In this case report, there is atresia of the lower esophagus at the esophagogastric junction. It appears to be an acquired event probably due to vascular compromise by the acute kinking of the stomach in the thorax because of the CDH. In an extensive review of the English literature, there is only one similar case reported by van Dooren et al. [5], where there was an atresia of the lower end of the esophagus that was not suspected until the postoperative period. In this case report, there was preoperative diagnosis of a lower esophageal obstruction; hence surgery was done using a chevron (rooftop) incision. This incision allows access for correction of both of the lesions. A feeding jejunostomy is a useful adjunct until the restoration of the esophagogastric continuity. 

In conclusion, esophageal anomalies are known to occur with CDH. Lower esophageal atresia is a rare association and a preoperative suspicion of this association, clinically, leads to timely correction of both of the lesions. A cheveron incision gives good access to correct both the CDH and lower esophageal atresia.

## Abbreviations

CDH: Congenital diaphragmatic hernia
GERD: Gastroesophageal reflux disease.
